# Childhood malnutrition and hypo mineralized molar defects: a cross sectional study

**DOI:** 10.12688/f1000research.74557.1

**Published:** 2021-12-22

**Authors:** Hoda Atef Abdelsattar Ibrahim, Rania Abdallah Nasr, Ahmed Adel Salama, Aya Ahmed Amin

**Affiliations:** 1 Lecturer, Pediatric Clinical Nutrition department, Pediatric department, Faculty of Medicine, Cairo University, Cairo University, Cairo, Cairo, 12613, Egypt; 2 Associate Professor , Pediatric Dentistry and Dental Public Health Department, Faculty of Dentistry, Cairo University., Cairo university, Cairo, Cairo, 12613, Egypt; 3 Lecturer, Pediatric Dentistry and Dental Public Health Department, Faculty of Dentistry, Cairo University., Cairo University, Cairo, Cairo, 12613, Egypt; 4 Assistant lecturer, Cancer Epidemiology and Biostatistics, National Cancer Institute, Cairo University, Cairo University, Cairo, Cairo, 12613, Egypt

**Keywords:** HSPM ;MIH ;dental caries ; Malnutrition; children

## Abstract

**Background**:  Malnutrition is well-known to yield high morbidities and mortalities and considering its consequence on the oral cavity, malnutrition is shown to have pre-eruptive and post-eruptive outcomes. The objective was to assess the prevalence of hypo-mineralized second primary molar (HSPM), molar–incisor hypo-mineralization (MIH) and dental caries in malnourished children as well as addressing the relation between types of malnutrition of the children and their dental morbidities represented in HSPM, MIH and dental caries.

**Methods** ***:*** This is a cross sectional analytical study. Malnourished children aged 5-10 years and presented to the Outpatient Clinic of Pediatric Dentistry Department, Faculty of Dentistry, Cairo University were examined for HSPM. MIH using European Academy of Pediatric Dentistry criteria and dental Caries using def/ DMF indices.

**Results** ***:***  A consecutive sample (a long six months) of 54 malnourished children were enrolled in the study. Dental caries was a greater dental morbidity in the overweight and obese group. Besides, stunting was a greater risk in HSPM and MIH. There was an association between HSPM and MIH in a considerable percentage of the study group.

**Conclusions** ***:*** Malnutrition is a risk factor for dental abnormalities. HSPM could expect the presence of MIH.

## Introduction

Malnutrition is an essential determinant of morbidity and mortality in young children. It is linked with 45 percent of all deaths in children below five years of age. ^1^^,^^2^ Malnutrition in children can include being underweight, being overweight, obesity, stunted growth and wasting. ^3^ Malnutrition and its subtypes have been linked to dental comorbidities. For example, hypo mineralization and dental caries have been linked to stunted growth and obesity respectively. In addition, co-existence between these dental disorders can occur. ^4^^-^^6^ Hypo mineralization represents the clinical existence of developmental defects which can be seen as discoloration, opacities or as a mixture of change in appearance and loss of enamel substance. ^7^ Presently, there has been increasing attention regarding the fact that hypo mineralization can be a sign of interruption in a child’s growth as a result of early childhood illnesses. ^8^^,^^9^

Currently, it has been addressed that a hypo-mineralized second primary molar (HSPM) could be a clinically important predictor for molar–incisor hypo-mineralization (MIH). ^10^

Dental caries is a multifactorial disease. Factors affecting the onset of this disease could include diet composition, oral hygiene and frequency, socioeconomic status, bacterial load, salivary immunoglobulins and fluoride intake. ^11^ It is determined as the single most common chronic disease during childhood. ^12^

Dental comorbidities can coexist together. The increased caries risk concomitant with hypo-mineralization results is a considerable dental morbidity that is often culminating in subsequent orthodontic consequences. ^13^

The study is aiming at assessing the dental abnormalities (HSPM, MIH, dental caries and co-occurrence of HSBM and MIH) in the malnourished children. The primary objective of the study is to detect the prevalence of HSPM, MIH, dental caries and co-occurrence of HSBM and MIH in malnourished children. Secondary objectives involve evaluating the association between HSPM, MIH, and dental caries with co-occurrence of HSBM and MIH, they also include assessing the association between the socioeconomic level, the type of malnutrition and dental abnormalities.

## Methods

### Study design

Observational cross sectional analytical study.

### Settings

Outpatient Clinic of Pediatric Dentistry Department, Faculty of Dentistry, Cairo University. The patient enrollment was from 1 ^st^ of April to end of September, 2021.

### Participants

***Eligibility criteria***

***Inclusion criteria*:** 1. Children aged from 5 to 10 years. 2. Both genders. 3. All children who were following in the dental clinic and were observed to be malnourished along the duration of the study (a long six months duration). 4. Children whose parents or caregivers agreed to be enrolled in the study.

***Exclusion criteria*:** 1. Children with extracted primary second molars and permanent incisors and molars. 2. Children with history of dental trauma. 3. Children with orthodontic bands or dental appliances. 4. Children whose parents or caregivers didn’t agree to be enrolled in the study.

### Methods of selection

1. A consecutive sample of malnourished children aged from 5 to 10 years attending the outpatient clinic in Pediatric Dentistry Department, Faculty of Dentistry, Cairo University were included in this study according to eligibility criteria.

Definitions of malnutrition in the study:

A. Underweight: when the weight for age is less than the mean by 2 standard deviations (SD) of the World Health Organization (WHO) Child Standards for growth or less than 3 ^rd^ centile for age.
B. Stunting: when the height for age is less than the mean by 2 standard deviations of the WHO Child Standards for growth in children. WHO percentiles can be used to address the stunted growth when it is less than 3 ^rd^ centile
C. Wasting: when Body Mass Index (BMI) is less than the 3 ^rd^ centile for age or BMI Z score is less than 2 SD of the WHO Child Standards for BMI 5-19 years.
D. Overweight: when Body Mass Index (BMI) is more than 85th centile for age or BMI z score is more than 1 SD of the WHO Child Standards for BMI 5-19 years.
E. Obesity: when BMI is more than 97th centile for age or BMI Z score is more than 2 SD of the WHO Child Standards for BMI 5-19 years.

2. Written informed consent was obtained from children parents or guardians accepting to participate in the study. Medical and sociodemographic data were recorded in the patient ^'^s chart.
3. Children were examined clinically on dental units using artificial light. Source of it was magnifying loop with led light. Flat mouth mirrors and sterilized standard no.4/6 double-ended exploration probes. Wet cotton swabs were used prior to the examination to remove excess plaque or saliva.
4. The diagnostic criteria for MIH and HSPM and scoring for them were established based on the European Academy of Pediatric Dentistry criteria ^7^^,^^8^
5. Caries Indices used in scoring dental caries; DMF (Permanent Teeth) where D = decayed indicated for restoration, M = missed due to caries, F = filled without recurrent caries. def (Primary teeth in mixed dentition) where d = decayed indicated for restoration, e= non-restorable indicated for extraction, f = filled without recurrent caries.
6. Socioeconomic status: using a valid assessment tool. Socioeconomic status was measured using a tool developed and validated by El-Gilany et al., 2012. It measures the socioeconomic level through seven domains, education and cultural, occupation, family, family possessions, economic, home sanitation and health care domains. Total score of the tool is ranging from 0 to 84, with higher scores indicating better socioeconomic status. ^14^

Sample size calculation

The sample size was calculated based on the primary objective in the study, which was detecting the prevalence of HSPM, MIH and dental caries in malnourished children **.** There was a previous study that estimated the prevalence of HSPM to be 5.6% and MIH to be 74%, ^15^ while another former one estimated the prevalence of dental caries to be 83.1% in malnourished children. ^16^ By setting the α as 0.05, power as 0.80, margin of error of 0.10 and the minimum required sample sizes for HSPM, MIH and dental caries is 21, 27 and 54 respectively. The largest sample size (54) was selected for the study.

***Efforts to address and avoid potential sources of bias***

We excluded pediatrics with associated chronic illness as they might have additional external factors for malnutrition due to the chronic disease effect.

***Data analysis***

All statistical calculations were performed using SPSS (Statistical Package for the Social Science; SPSS Inc., Chicago, IL, USA) version 25, Categorical data was statistically described in terms of frequencies and percentages, while quantitative data were described in terms of mean and standard deviation, median, interquartile range and range as appropriate. Comparison of quantitative variables between the different groups of malnutrition was done using Kruskal-Wallis test as the variables were not normally distributed. Post hoc pairwise comparisons with Bonferroni adjustment of p value were performed between the groups. For comparing categorical data Chi square (χ ^2^) test was performed, but obtaining p value was not applicable as 7 cells (> 25%) have expected count less than 5. For performing the correlation between two numerical variables, Spearman rho correlation was performed as the variables were not normally distributed.

***Ethical considerations***

The study was reviewed and approved by the scientific research committee and ethics of Cairo University, Faculty of Dentistry (ethical clearance number, 13321) and the study was carried out in accordance with Cairo University's laws human research. Throughout the study, the privacy and confidentiality of the data were preserved, the results were presented anonymously without disclosure of patients’ personal identifying information. Written informed consent for participation in the study and publishing was taken.

## Results

### Demographic criteria

This study is a cross-sectional one which enrolled pediatric patients who were following in the outpatient dentist clinic across the duration of the study and observed to be malnourished for addressing the prevalence of HSPM, MIH and dental caries.

Total numbers of the study participants enrolled were 54 patients. The mean age was 7.10 ± 1.34. 50 % (n = 27) were males and 50% (n = 27) were females with male to female ratio 1:1 ( Table 1).

**Table 1.** Demographic criteria of the study participants.

Characteristics	Number (n = 54)	Percent (%)
**Age groups (years)**		
**5–<7**	24	44.4
**7–10**	30	55.6
**Mean ± SD** *	7.1 ± 1.4	
**Range**	5.0–9.6	
**Gender**		
**Male**	27	50.0
**Female**	27	50.0
**Male: Female ratio**	1: 1	

* SD: Standard Deviation.

Prevalence of dental abnormalities (HSPM, MIH and dental caries) in the malnourished enrolled children were assessed ( Table 2). It was 92.6% (n = 50) of the study group.

**Table 2.** Prevalence of different dental abnormalities in malnourished children.

Dental abnormalities	Number	Percent	95% confidence interval
Prevalence of HSPM for primary teeth (n = 53) *	25	47.2	(33.3–61.4)
Prevalence of MIH for permanent teeth (n = 42) *	19	45.2	(29.8–61.3)
Prevalence of dental caries for primary teeth (n = 53) *	44	83.0	(70.2–91.9)
Prevalence of dental caries for permanent teeth (n = 42) *	72	64.3	(48–78.4)
Prevalence of dental abnormalities (HSPM or MIH or Dental caries) (n = 54) *	50	92.6	(82.1–97.9)

* The remaining cases (total number 54) were not applicable.

For HSPM, its prevalence in malnourished children was 47.2% (n = 25) of the study participants. Regarding MIH, its prevalence was 45.2% (n = 19) of the malnourished enrolled study group. Lastly, the prevalence for dental caries was 64.3 % (n = 72) in the permanent teeth and 83.0 % (n = 44) in the primary teeth.

There was a co-occurrence between hypo-mineralized second primary molar and molar incisor hypo mineralization in the study group. It was 39% (n = 16) of the study group ( Table 3).

**Table 3.** Co-occurrence between HSPM and MIH in the enrolled study group.

Co-occurrence of HSPM and MIH (n = 41)	Number	Percent	95% confidence interval
Yes	16	39.0	24.2–55.5
No	25	61.0	44.5–75.8

The associations between the co-occurrence of HSPM and MIH and the HSPM, MIH and dental caries were examined. There were significant differences in the median HSPM for primary teeth, median MIH for permanent teeth and median CI (caries index) for primary teeth between patients who had co-occurrence and patients who didn’t. Median HSPM for primary teeth was 16.6% in patients who had co-occurrence of HSPM and MIH compared to 0% in patients who didn’t have this co-occurrence. Median MIH for permanent teeth was 20% in patients who had co-occurrence of HSPM and MIH compared to 0% in patients who didn’t. Also, CI for primary teeth was 7.4 in patients who had co-occurrence of HSPM and MIH compared to 4 in patients who didn’t ( Table 4).

**Table 4.** The association between the co-occurrence of HSPM and MIH; and different dental abnormalities.

	Co-occurrence of HSPM and MIH		P value **
	Yes (n = 16)	No (n = 25)	
	Median (IQR) * Min–Max	Median (IQR) * Min–Max	
**HSPM for primary teeth (%)**	16.6 (13.2–19.4)	0.0 (0.0–0.0)	< 0.001 **
	0.14–25.0	0.0–11.0	
**MIH for permanent teeth (%)**	20.0 (16.6–25.0)	0.0 (0.0–0.0)	< 0.001 **
	8.0–50.0	0.0–33.0	
**CI for primary teeth**	7.5 (4.5–10.0)	4.0 (0.0–7.0)	< 0.001 **
	0.0–13.0	0.0–11.0	
**CI for permanent teeth**	2.0 (0.3–4.0)	1.0 (0.0–3.0)	0.248
	0.0–9.0	0.0–6.0	

* IQR: Interquartile Range.

** P value is significant if < 0.05.

Malnutrition in the enrolled children was assessed **.** The overweight group represented 24.1 % (n = 13). The obese group represented 16.7 % (n = 9). The underweight group represented 11.1 % (n = 6). The wasted group represented 18.5 (10 %). The stunted group represented 22 % (n = 40.7%) ( Table 5).

**Table 5.** Types of malnutrition in the study participants.

Type of malnutrition	Number (n = 54)	Percent (%) *
Overweight	13	24.1
Obese	9	16.7
Underweight	6	11.1
Wasted	10	18.5
Stunted	22	40.7

* Some cases showed more than one malnutrition disorder.

Table 6 shows the associations between the types of malnutrition and the dental examination outcomes . Both HSPM and the MIH were significantly different among the types of malnutrition. For the HSPM, by performing post hoc pairwise comparisons with Bonferroni adjustment to the P value, it was found that HSPM was significantly different between the stunted group (median HSPM of 14.2%) and the overweight or obese group (median HSPM of 0.0%) (P value 0.01). Regarding the MIH level, by performing post hoc pairwise comparisons with Bonferroni adjustment to the P value, there were significant differences between the stunted group (median MIH of 19.4%) and overweight or obese group (median MIH of 0.0%) (P value 0.001), as well as between the stunted group (median MIH of 19.4%) and wasted groups (median MIH of 0.0%) (P value 0.025).

**Table 6.** Correlation between different types of malnutrition and dental examination outcomes.

Type of malnutrition	HSPM for primary teeth (%)	MIH for permanent teeth (%)	CI for primary teeth	CI for permanent teeth	Co-occurrence of HSPM and MIH
	Median (IQR) *	Median (IQR) *	Median (IQR) *	Median (IQR) *	n (%) ***
Overweight or obese	0 (0–6.7)	0 (0–0)	6 (4–8.5)	2 (0–4.5)	3 (18.8)
Stunted	14.2 (0–16.9)	19.4 (15.2–25)	6 (3–10.8)	1 (0–3.3)	10 (71.4)
Underweight and stunted	5.6 (0–16.5)	20 (0–33.3)	5.5 (0–10.3)	1 (0–3.5)	2 (40.0)
Wasted	0 (0–8.7)	0 (0–4.2)	2 (0–7.8)	1.5 (0–3.5)	16 (39)
P value **	0.011 **	< 0.001 **	0.234	0.658	Not applicable ****

* IQR: Interquartile Range.

** P value is significant if < 0.05.

*** The number and percentages of cases that showed the prevalence of co-occurrence of HSPM and MIH within each category of malnutrition.

**** Chi square test is not applicable as 7 cells (> 25%) have expected count less than 5.

Correlations between dental abnormalities in the study group were assessed. MIH for permanent teeth showed a significant weak to moderate direct correlation with CI for primary teeth. HSPM showed a weak direct correlation with CI for primary teeth with borderline significance ( Table 7).

**Table 7.** Correlation between hypo mineralized secondary primary molar, molar incisor hypo mineralization and dental caries

		CI for primary teeth	CI for permanent teeth
**HSPM for primary teeth (%)**	**Spearman rho correlation**	r = 0.248	r = 0.055
	**P: value** *	0.073	0.734
**MIH for permanent teeth (%)**	**Spearman rho correlation**	r = 0.357	r = 0.105
	**P: value** *	0.022 *	0.508

* P value is significant if < 0.05.

The Socioeconomic level of the study participants was investigated ( Table 8).

**Table 8.** The socioeconomic level in the study group.

Socioeconomic status (Score = 0–84)	Number (n = 54)	Percent (%)
Very low (0–37)	12	22.2
Low (38–47)	19	35.2
Moderate (48–58)	11	20.4
High (59–84)	12	22.2
Total Socioeconomic Score Median (IQR) *	43 (37–57.3)	

* IQR: Interquartile Range.

The most common socioeconomic level was the low one.

The associations between types of malnutrition of the enrolled children and their socioeconomic levels were investigated ( Table 9). The highest score of the socioeconomic level was among the obese patients.

**Table 9.** The association between the socioeconomic level of the enrolled children and their malnutrition.

Type of malnutrition	Socioeconomic Score Median (IQR) *
Overweight or obese	49.5 (42–62.3)
Stunted	39.5 (34–53.8)
Underweight and stunted	43 (37.5–54.3)
Wasted	43 (32–48.5)
P value **	0.142

* IQR: Interquartile Range.

** P value is significant if < 0.05.

The association between the socioeconomic levels of the study group and the different dental abnormalities was investigated. HSPM was significantly different among the socioeconomic levels. By performing post hoc pairwise comparisons with Bonferroni adjustment to the P value, the HSPM showed a statistically significant difference between the very low group (median HSPM of 12.9%) and the high group (median HSPM of 0.0%) with P value of 0.017 ( Table 10).

**Table 10.** Correlations between socioeconomic levels and different dental abnormalities

Socio-economic level	HSPM for primary teeth (%)	MIH for permanent teeth (%)	CI for primary teeth	CI for permanent teeth
	Median (IQR) ** Min–Max	Median (IQR) ** Min–Max	Median (IQR) ** Min–Max	Median (IQR) ** Min–Max
Very low	12.9 (0.0–17.4)	12.1 (0.0–17.5)	4.0 (1.3–6.8)	2.0 (0.0–4.0)
	0.0–25.0	0.0–50.0	0.0–18.0	0.0–9.0
Low	7.1 (0.0–11.1)	0.0 (0.0–20.0)	7.0 (2.0–10.0)	2.0 (0.0–3.0)
	0.0–20.0	0.0–33.0	0.0–13.0	0.0–8.0
Moderate	0.0 (0.0–11.3)	0 (0.0–20.0)	7.5 (5.8–9.3)	3.0 (1.0–4.5)
	0.0–20.0	0.0–25.0	0.0–10.0	0.0–5.0
High	0.0 (0.0–0.0)	0.0 (0.0–8.3)	6.0 (2.0–8.5)	0.0 (0.0–3.3)
	0.0–17.0	0.0–25.0	0.0–12.0	0.0–5.0
P value *	0.019 *	0.627	0.606	0.301

* P value is significant if < 0.05.

** IQR: Interquartile Range.

## Discussion

This study is a cross sectional one which enrolled malnourished pediatric patients aged 5-10 years old who were following at dentist clinic at Cairo University for assessing the prevalence of HSPM, MIH and dental caries.

Regarding the association between obesity or overweight group and the caries indices, our results agree with a previous one in which sensitivity analyses revealed that the obese children had more caries in their primary teeth than the normal-weight children. Considerably, more caries was noticed among the overweight and obese group in both the primary and the permanent teeth. ^17^

Regarding the association between MIH and the stunted growth, our results disagree with a previous study which showed that stunted schoolchildren had no significant correlation or association with MIH. ^18^ This disagreement may be due to the high prevalence of short stature in the Egyptian children. ^19^^,^^20^

Regarding the association between socioeconomic level and obesity in the study participants, our results don’t agree with a previous study and found the higher prevalence of obesity in children with low socioeconomic levels. This different result may be due to different cultures. ^21^

Regarding the co-occurrence between HSPM and MIH, the present study goes in line with a former one that yielded similar results. That study concluded that HSPM can be considered a predictor of MIH. ^22^

Regarding the association between HSPM and caries index, HSPM showed a weak direct correlation with CI for primary teeth with borderline significance. This result is very close to a previous one in which an increased incidence of dental caries was found in children with HSPM. ^23^

Regarding the association between MIH and caries index, the present study shows a significant correlation. This result agrees with a previous study which yielded that caries values are much higher in children with MIH. However, it disagrees with it in that the total prevalence of MIH in that study was low unlike our study which showed a high prevalence of MIH as the study participants were malnourished. This reveals that malnutrition is a considerable risk for MIH. ^24^

Regarding the association between socioeconomic levels and the dental abnormalities, the present study showed higher percentages of MIH in lower socioeconomic levels. These results agree with a previous study which yielded lower socioeconomic levels in MIH. ^25^ The present study slightly disagrees with a previous one in which caries index was higher in study groups with low socioeconomic levels and in which the socioeconomic level contributed no significant risk for HSPM. ^26^ The latter disagreement may be due that in this study, the malnutrition was an added risk factor beside the low socioeconomic level.

### Study limitations

Not including controls of well-nourished children is considered as a limitation of the study. Not including controls of well nourished children in the study may make it impossible to detect whether the dental abnormalities are more prevalent in the malnourished children or not. Also, the non-probability sampling technique (consecutive sampling method) may compromise the generalizability (the external validity of the study) because it makes the study more liable for selection bias.

## Conclusion

Malnutrition could be a risk factor for dental abnormalities. Besides, children with low socioeconomic levels have a greater incidence for MIH. In addition, different dental abnormalities could co-exist together. Screening for HSPM, MIH and CI in malnourished children would be a welcome development.

## Data availability

### Underlying data

Figshare. Childhood malnutrition and Hypomineralized molar defects, a cross sectional study. https://doi.org/10.6084/m9.figshare.16778557.v2. ^27^

Data are available under the terms of the Creative Commons Zero “No rights reserved” data waiver (CC0 1.0 Public domain dedication).
