# Childhood malnutrition and hypo mineralized  molar defects ;a cross sectional study, Egypt

**DOI:** 10.12688/f1000research.74557.2

**Published:** 2022-02-14

**Authors:** Hoda Atef Abdelsattar Ibrahim, Rania Abdallah Nasr, Ahmed Adel Salama, Aya Ahmed Amin

**Affiliations:** 1 Lecturer, Pediatric Clinical Nutrition department, Pediatric department, Faculty of Medicine, Cairo University, Cairo University, Cairo, Cairo, 12613, Egypt; 2 Associate Professor , Pediatric Dentistry and Dental Public Health Department, Faculty of Dentistry, Cairo University., Cairo university, Cairo, Cairo, 12613, Egypt; 3 Lecturer, Pediatric Dentistry and Dental Public Health Department, Faculty of Dentistry, Cairo University., Cairo University, Cairo, Cairo, 12613, Egypt; 4 Assistant lecturer, Cancer Epidemiology and Biostatistics, National Cancer Institute, Cairo University, Cairo University, Cairo, Cairo, 12613, Egypt

**Keywords:** Hypomineralized Second Primary Molars (HSPM); MIH; dental caries; Malnutrition; children

## Abstract

**Background**:  Malnutrition is well-known to yield high morbidities and it has pre-eruptive and post-eruptive consequences. The objective of the study was to evaluate the prevalence of Hypomineralized Second Primary Molars (HSPM), Molar Incisor Hypomineralization (MIH) and dental caries in malnourished children, as well as addressing the relation between types of malnutrition and the dental morbidities.

**Methods:** This is a cross sectional analytical study. A consecutive sample of 54 malnourished cases aged 5-10 years and presented to the Outpatient Clinic of Pediatric Dentistry Department, Faculty of Dentistry, Cairo University across 6 months period were examined for HSPM, MIH – using the  European Academy of Pediatric Dentistry criteria - and dental Caries using def/ DMF indices. Outcomes were the presence or absence of MIH and HSPM and their levels measured as percentage as well as Caries Indices. Exposures were types of malnutrition and the socioeconomic status.

**Results *:***  The mean age of study participants was 7.10 ± 1.34 years. HSPM and MIH were found in 47.2% and 45.2% of the study participants respectively, while dental caries was observed in 83% and 64.3% for primary and permanent teeth respectively. There was co-occurrence between HSPM and MIH in 39% of the cases.

HSPM level was significantly different in various types of malnutrition. It showed significant difference between the stunted group (median HSPM of 14.2%) and the overweight or obese group (median HSPM of 0.0%) (P value 0.01). MIH level showed significant differences between the stunted group (median MIH of 19.4%) and overweight or obese group (median MIH of 0.0%) (p value 0.001), as well as between the stunted group (median MIH of 19.4%) and wasted group (median MIH of 0.0%) (p value 0.025).

**Conclusions *:*** Malnourished children have high prevalence of dental abnormalities. HSPM could expect the presence of MIH.

## Introduction

Malnutrition is an essential determinant of morbidity and mortality in young children. It is linked with 45 percent of all deaths in children below five years of age. ^1, 2^ Malnutrition in children can include being underweight, being overweight, obesity, stunted growth and wasting; this classification is based on WHO definitions for malnutrition. ^3, 4^ Malnutrition and its subtypes have been linked to dental comorbidities. For example, hypo mineralization and dental caries have been linked to stunted growth and obesity respectively. In addition, co-existence between these dental disorders can occur. ^5- 7^

Hypomineralization represents the clinical existence of developmental flaws which can be detected as discoloration, opacities or as a combination of changes in appearance and loss of the enamel material. ^8^ Presently, there has been growing attention about the fact that hypo mineralization can be a mark of interruption in a child’s growth as a result of early childhood illnesses. ^9, 10^ Currently, it has been addressed that a hypo-mineralized second primary molar (HSPM) could be a clinically significant predictor for molar incisor hypomineralization (MIH). ^11, 12^

Clinically, defects of Molar Incisor Hypomineralization exist as opaque lesions with colors that are varied from white to yellow or brown, with distinctive margins between the affected and the sound enamel. In severe lesions, post-eruptive enamel breakdown could exist rapidly after erupting of the tooth in the oral cavity presenting as enamel hypoplasia rather than hypomineralization. Pediatrics affected with these defects have greater treatment requirements ^13, 14^ and had revealed more dental fear and anxiety ^15^ that further complicated the therapy. The requirement for the orthodontic therapy intervention as a result of tooth extraction induced by MIH has also been stated. ^16, 17^

Dental caries is a multifactorial disease. Factors affecting the onset of this disease could involve diet composition, oral hygiene, socioeconomic status, bacterial load, salivary immunoglobulins and fluoride intake. It is determined as the single most common chronic disease during childhood. ^18^

Obesity (or overweight) and dental caries are mounting problems regards the public health. Both obesity and dental caries are thought out to be greatly widespread, chronic, and share multifactorial circumstances, with potentially and significant lifelong influences on the lives of children and young people. ^19- 22^ The two problems are considered to share common causative influences, including dietary, biological, genetic, socioeconomic, cultural and environmental factors. ^23, 24^ Subsequently, a correlation between obesity and dental caries appears reasonable. More information about this association may allow the development of more efficient and effective targeted initiatives of the public health to diminish the incidence of obesity and dental caries. ^25, 26^

Dental comorbidities can coexist together. The increased caries possibility concomitant with hypo-mineralization results is a considerable dental morbidity that is often culminating in consequent orthodontic consequences. ^27^

This study is aiming at assessing the dental abnormalities (HSPM, MIH, dental caries and co-occurrence of HSPM and MIH) in the malnourished children. The primary objective of this study is to estimate the prevalence of HSPM, MIH, dental caries and co-occurrence of HSPM and MIH in the malnourished children. Secondary objectives involve assessing the association between HSPM, MIH, and dental caries with co-occurrence of HSPM and MIH and determining the link between the socioeconomic level, the type of malnutrition and dental abnormalities.

## Methods

### Study design

Observational cross sectional analytical study.

### Settings

Outpatient Clinic of Pediatric Dentistry Department, Faculty of Dentistry, Cairo University. The patient enrollment was from 1 ^st^ of April to end of September, 2021.

### Participants

***Eligibility criteria:***

***Inclusion criteria*:** 1. Children aged from 5 to 10 years. 2. Both genders. 3. All children who were following in the dental clinic and was observed to be malnourished along the duration of the study (a long six months duration). 4. Children whose parents or caregivers agreed to be enrolled in the study.

***Exclusion criteria*:** 1. Children with removed primary second molars and permanent molars and incisors. 2. Children with any history of dental trauma. 3. Children with dental appliances or orthodontic bands. 4. Children whose parents or caregivers didn’t agree to be enrolled in the study. 5. Children with chronic illness such as chronic digestive diseases.

### Methods of selection

A consecutive sample of 54 malnourished children aged from 5 to 10 years presenting at the outpatient clinic in Pediatric Dentistry Department, Faculty of Dentistry, Cairo University were enrolled in this study according to the mentioned eligibility criteria.

Definitions of malnutrition in the study: (3)

A. Underweight: when the weight for age is ˂ the mean by 2 standard deviations (SD) of the World Health Organization (WHO) Child Standards for growth or less than 3 ^rd^ centile for the age.
B. Stunting: when the height for age is ˂ the mean by 2 standard deviations of the WHO Child Standards for growth in children. WHO percentiles can be used to address the stunted growth when it is less than 3 ^rd^ centile.
C. Wasting: when BMI is ˂ than the 3 ^rd^ centile for age or BMI Z score is less than 2 SD of the WHO Child Standards for BMI 5-19 years.
D. Overweight: when BMI is ˃ 85th centile for age or BMI z score is more than 1 SD of the WHO Child Standards for BMI 5-19 years.
E. Obesity: when BMI is more than 97th centile for age or BMI Z score is more than 2 SD of the WHO Child Standards for BMI 5-19 years.

Written informed consent was obtained from parents or guardians accepting to participate in the present study. Medical and sociodemographic data were documented in the patient ^'^s chart. Children were observed clinically on dental units using artificial light. Source of it was magnifying loop with led light. Wet cotton swabs were used prior to the examination to remove excess plaque or saliva. The diagnostic criteria for MIH and HSPM and scoring for them were established based on the European Academy of Pediatric Dentistry criteria. ^8, 9^ Caries Indices used in scoring dental caries; DMF (Permanent Teeth) where D = decayed indicated for restoration, M= missed due to caries, F= filled without recurrent caries. def (Primary teeth in mixed dentition) where d = decayed indicated for restoration, e= non-restorable indicated for extraction, f= filled without recurrent caries. Socioeconomic status: using a valid assessment tool. Socioeconomic status was measured using a tool developed and validated by El-Gilany et al., 2012. It measures the socioeconomic level through seven domains, education and cultural, occupation, family, family possessions, economic, home sanitation and health care domains. Total score of the tool is ranging from 0 to 84, with higher scores indicating better socioeconomic status. ^28^

Sample size calculation:

The sample size was estimated based on the primary objective in the study, which was detecting the prevalence of HSPM, MIH and dental caries in malnourished children. There was a previous study that estimated the prevalence of HSPM to be 5.6% and MIH to be 74%, ^29^ while another former one estimated the prevalence of dental caries to be 83.1% in malnourished children. ^30^ By setting the α as 0.05, power as 0.80, margin of error of 0.10, the minimum required sample sizes for estimating the prevalence of HSPM, MIH and dental caries are 21, 27 and 54 respectively. The largest sample size (54) was selected for the study.

***Efforts to address and avoid potential sources of bias:***

We ruled out pediatrics with associated chronic illness as they could have additional issues regards malnourishment due to the long-lasting course effect.

***Data analysis***

All statistical calculations were completed using SPSS (Statistical Package for the Social Science; SPSS Inc., Chicago, IL, USA) version 25, Categorical data were statistically set out in terms of frequencies and percentages, while quantitative data were described in terms of mean and standard deviation, median, interquartile range and range as appropriate. Dental abnormalities were compared between patients who have co-occurrence between HSPM and MIH and patients who do not using Mann-Whitney U test as numerical non-normally distributed variable. Comparison of dental abnormalities between different groups of malnutrition was done using Kruskal-Wallis test as the variables were not normally distributed. Post hoc pairwise comparisons with Bonferroni adjustment of p value were performed between the groups. For comparing categorical data Chi square (χ ^2^) test was done, but obtaining p value was not applicable as 7 cells (> 25%) have expected count less than 5. For performing the correlations between HSPM and MIH with Caries Indices, Spearman's rho correlation was performed as they were not normally distributed.

***Ethical considerations***

The study was reviewed and approved by the scientific research committee and ethics of Cairo University, Faculty of Dentistry (ethical clearance number, 13321) and the study was carried out in an agreement with Cairo University's laws for human research. Throughout the study, the privacy and confidentiality of the data were preserved, the results were presented anonymously without disclosure of patients’ personal identifying information. Written informed consent for participation in the study and publishing was taken.

## Results

The study included 54 malnourished pediatric patients. The mean age of study participants was 7.10 ± 1.34 years, ranging from 5 to 9.6 years. 50 % of the patients (n = 27) were males and 50% were females.

Prevalence of dental abnormalities (HSPM, MIH and dental caries) in the enrolled children were assessed ( Table 1). 92.6% (n = 50) of the study participants had at least one type of dental abnormality.

**Table 1.** Prevelance of different dental abnormalities in malnourished children (n=54).

Dental abnormalities	Number	Percent	95% Confidence Interval
Prevalence of HSPM for primary teeth (n=53) *	25	47.2	(33.3 – 61.4)
Prevalence of MIH for permanent teeth (n=42) *	19	45.2	(29.8 – 61.3)
Prevalence of dental caries for primary teeth (n=53) *	44	83.0	(70.2 – 91.9)
Prevalence of dental caries for permanent teeth (n=42) *	27	64.3	(48 – 78.4)
Prevalence of dental abnormalities (HSPM or MIH or Dental caries) (n=54) *	50	92.6	(82.1 – 97.9)
Co-occurrence of HSPM and MIH (n=41)			
Yes	16	39.0	24.2 – 55.5
No	25	61.0	44.5 – 75.8

* The remaining cases (total number 54) were not applicable.

For HSPM, its prevalence in malnourished children was 47.2% (n= 25) while MIH was detected in 45.2% (n= 19) of the study participants. Dental caries was more prevalent, it was found in 64.3 % (n= 27) for the permanent teeth, and in 83.0 % (n= 44) for the primary teeth. Co-occurrence between HSPM and MIH was observed in 39% (n= 16) of the study group.

The associations between the co-occurrence of HSPM and MIH with the HSPM, MIH and dental caries were examined. There were significant differences in the median HSPM for primary teeth, median MIH for permanent teeth and median CI (caries index) for primary teeth between patients who had co-occurrence and patients who didn’t. Median HSPM for primary teeth was 16.6% in patients who had co-occurrence of HSPM and MIH compared to 0% in patients who didn’t have this co-occurrence. Median MIH for permanent teeth was 20% in patients who had co-occurrence of HSPM and MIH compared to 0% in patients who didn’t. Besides, CI for primary teeth was 7.5 in patients who had co-occurrence of HSPM and MIH compared to 4 in patients who didn’t ( Table 2).

**Table 2.** The associations between co-occurrence of HSPM and MIH; and different dental abnormalities in malnourished children (n=41).

	Co-occurrence of HSPM and MIH		P value **
	Yes (n=16)	No (n=25)	
	Median (IQR) * Min - Max	Median (IQR) * Min - Max	
**HSPM for primary teeth (%)**	16.6 (13.2 – 19.4)	0.0 (0.0 – 0.0)	< 0.001 **
	0.14 – 25.0	0.0 – 11.0	
**MIH for permanent teeth (%)**	20.0 (16.6 – 25.0)	0.0 (0.0 – 0.0)	< 0.001 **
	8.0 – 50.0	0.0 – 33.0	
**CI for primary teeth**	7.5 (4.5 – 10.0)	4.0 (0.0 – 7.0)	< 0.001 **
	0.0 – 13.0	0.0 – 11.0	
**CI for permanent teeth**	2.0 (0.3 – 4.0)	1.0 (0.0 – 3.0)	0.248
	0.0 – 9.0	0.0 – 6.0	

* IQR: Interquartile Range.

** p value is significant if < 0.05.

Types of malnutrition in the enrolled children were assessed **.** The overweight group represented 24.1% (n=13) of the cases while obese participants represented 16.7% (n=9). The underweight group was 11.1% (n=6) of the patients. The wasted children were 18.5% (n=10), while 40.7% of the cases (n=22) showed stunting. Some cases showed more than one type malnutrition.

Table 3 shows the associations between the types of malnutrition and the dental examination outcomes. Both HSPM and the MIH were significantly different among the types of malnutrition. For the HSPM, by performing post hoc pairwise comparisons with Bonferroni adjustment to the P value, it was found that HSPM was significantly different between the stunted group (median HSPM of 14.2%) and the overweight or obese group (median HSPM of 0.0%) (P value 0.01). Regarding the MIH level, by performing post hoc pairwise comparisons with Bonferroni adjustment to the P value, there were significant differences between the stunted group (median MIH of 19.4%) and overweight or obese group (median MIH of 0.0%) (p value 0.001), as well as between the stunted group (median MIH of 19.4%) and wasted groups (median MIH of 0.0%) (P value 0.025).

**Table 3.** Correlation between different types of malnutrition and dental abnormalities in malnourished children (n=54).

Type of Malnutrition	HSPM for primary teeth (%)	MIH for permanent teeth (%)	CI for primary teeth	CI for permanent teeth	Co-occurrence of HSPM and MIH
	Median (IQR) *	Median (IQR) *	Median (IQR) *	Median (IQR) *	n (%) ***
Overweight or obese	0 (0 – 6.7)	0 (0 – 0)	6 (4 – 8.5)	2 (0 – 4.5)	3 (18.8)
Stunted	14.2 (0 – 16.9)	19.4 (15.2 – 25)	6 (3 – 10.8)	1 (0 – 3.3)	10 (71.4)
Underweight and stunted	5.6 (0 – 16.5)	20 (0 – 33.3)	5.5 (0 – 10.3)	1 (0 – 3.5)	2 (40.0)
Wasted	0 (0 – 8.7)	0 (0 – 4.2)	2 (0 – 7.8)	1.5 (0 – 3.5)	16 (39)
P value **	0.011 **	< 0.001 **	0.234	0.658	Not applicable ****

* IQR: Interquartile Range.

** p value is significant if < 0.05.

*** The number and percentages of cases that showed the prevalence of co-occurrence of HSPM and MIH within each category of malnutrition.

**** Chi square test is not applicable as 7 cells (> 25%) have expected count less than 5.

Correlations between dental abnormalities in the study group were assessed. MIH for permanent teeth showed a significant weak to moderate direct correlation with CI for primary teeth. HSPM showed a weak direct correlation with CI for primary teeth with borderline significance ( Table 4).

**Table 4.** Correlation between dental caries with hypo-mineralized second primary molar and molar–incisor hypo-mineralization in malnourished children (n=54).

		CI for primary teeth	CI for permanent teeth
**HSPM for primary teeth (%)**	**Spearman rho correlation**	r = 0.248	r = 0.055
	**P: value** *	0.073	0.734
**MIH for permanent teeth (%)**	**Spearman rho correlation**	r = 0.357	r = 0.105
	**P: value** *	0.022 *	0.508

* P value is significant if < 0.05.

The socioeconomic level of the study participants was investigated. The median socioeconomic score of study participants was 43 (Interquartile range: 37 – 57.3) on a scale ranging from 0 to 84. One third of the patients (35.2%) were from the low socioeconomic level. 22.2% of them had very low socioeconomic level, 20.4% were of moderate level, and 22.2% of the cases had high socioeconomic level.

Associations between types of malnutrition of the enrolled children and their socioeconomic levels were assessed ( Table 5). The highest score of socioeconomic level was among the obese patients.

**Table 5.** The association between the socioeconomic level of the enrolled children and their malnutrition in malnourished children attending outpatient clinic of pediatric dentistry department, Cairo University (N=54).

Type of Malnutrition	Socioeconomic Score Median (IQR) *
Overweight or obese	49.5 (42 – 62.3)
Stunted	39.5 (34 – 53.8)
Underweight and stunted	43 (37.5 – 54.3)
Wasted	43 (32 – 48.5)
P value **	0.142

* IQR: Interquartile Range.

** P value is significant if < 0.05.

Regarding the associations between socioeconomic levels of the study group and different dental abnormalities; HSPM was significantly different among the socioeconomic levels. By performing post hoc pairwise comparisons with Bonferroni adjustment to the P value, the HSPM showed a statistically significant difference between the very low group (median HSPM of 12.9%) and the high group (median HSPM of 0.0%) with p value of 0.017 ( Table 6).

**Table 6.** Correlations between socioeconomic levels and different dental abnormalities in malnourished children attending outpatient clinic of pediatric dentistry department, Cairo University (N=54).

Socio-Economic level	HSPM for primary teeth (%)	MIH for permanent teeth (%)	CI for primary teeth	CI for permanent teeth
	Median (IQR) ** Min - Max	Median (IQR) ** Min - Max	Median (IQR) ** Min - Max	Median (IQR) ** Min - Max
Very Low	12.9 (0.0 – 17.4)	12.1 (0.0 – 17.5)	4.0 (1.3 – 6.8)	2.0 (0.0 – 4.0)
	0.0 – 25.0	0.0 – 50.0	0.0 – 18.0	0.0 – 9.0
Low	7.1 (0.0 – 11.1)	0.0 (0.0 – 20.0)	7.0 (2.0 – 10.0)	2.0 (0.0 – 3.0)
	0.0 – 20.0	0.0 – 33.0	0.0 – 13.0	0.0 – 8.0
Moderate	0.0 (0.0 – 11.3)	0 (0.0 – 20.0)	7.5 (5.8 – 9.3)	3.0 (1.0 – 4.5)
	0.0 – 20.0	0.0 – 25.0	0.0 – 10.0	0.0 – 5.0
High	0.0 (0.0 – 0.0)	0.0 (0.0 – 8.3)	6.0 (2.0 – 8.5)	0.0 (0.0 – 3.3)
	0.0 – 17.0	0.0 – 25.0	0.0 – 12.0	0.0 – 5.0
P value *	0.019 *	0.627	0.606	0.301

* P value is significant if < 0.05.

** IQR: Interquartile Range.

## Discussion

The nutritional status of pediatrics has an effect on their development and health. Thus, the physical, mental, social, and nutritional status of them, as well as the other features that are related to malnutrition, should be assessed periodically to monitor malnutrition, hence, suitable protective measures can be enabled. ^31^

In our study, the obese or overweight children occupied a great percent of the study group. In fact, the economic burden of the country could be a helping factor. To illustrate, Mowafi, et al. 2014 has discussed the socioeconomic status – obesity association (SES – obesity). Interestingly, the developing countries occupy positive SES – obesity associations. This means that obesity is more commonly found in individuals with higher socioeconomic levels – as our study yielded – in contrast to the developed countries. Individuals with low SES in the developing countries might be further restricted in their capability of calories diversion with greater -nutrient foods, such as meats, fruits and vegetables. By comparison, humans from higher SES categories may be capable of accessing more both increased quality and quantity of foods, potentially resulting in high levels of overweight and obesity as well. ^32^ Another explanation is the nutrition transition. It states the interaction of demographic, economic, environmental and cultural alterations in a society which are linked to shifting patterns of the nutritional intakes. ^33- 36^ In general, countries or societies with poorer ranks of economic development such as Egypt are earlier in the nutrition transition and display a positive SES-obesity relationship among the individuals. Those societies which are more developed tend to exhibit an inverse SES-obesity association among their individuals. ^37^ This can explain why our findings don’t match with the findings of Yang, et al. 2019 who found the higher prevalence of obesity in children with the low socioeconomic levels. ^38^

Unfortunately, obesity or overweight has undesirable dental associations. It is linked to great risks of the caries indices in the current research. A systematic review done by Chen, et al 2018, revealed similar results in which sensitivity analyses showed that the obese children had more caries in their primary teeth than the normal-weight children. Considerably, more caries was noticed among the overweight and obese group in the primary and the permanent teeth. ^39^ Other studies have also found positive associations between caries and BMI. ^40, 41^

Another parallel result was found in a report carried out by Serdar, et al, 2020 who observed positive correlation between obesity and DMFT index (differences in DMFT index among BMI groups was detected to be statistically significant (p = 0.001; p < 0.01). DMFT index was significantly greater in the obese group than in the normal-weight group (p = 0.001) and overweight group (p = 0.001). That study recommended co-administration of the obesity prevention programs and the preventive oral health programs that can enhance the public health to a better point. ^42^ Evidence of a link between BMI (Body Mass Index) and caries was inconsistent. Based on the researches with lower likelihood of being flawed, a positive correlation between the variables of interest was observed chiefly in older children. In younger kids, the evidence was equivocal. Longitudinal studies investigating the correlations or links between different indicators of obesity and caries over the life course will aid in understanding their complex link. ^43^

The link between BMI and dental caries in pediatrics is more complicated than what could be clarified by the carbohydrate intake alone. ^44^ As it is well recognized, dental caries is a disease resulted from numerous risk factors such as oral habits, microorganisms, genetics, and fluoride treatment; so the sole factor of diet might be enhanced or flattened by other factors. ^45^ Qomsan et al., 2017 suggested a similar opinion. It was concluded that high intake of free sugar is a well-established risk factor for the dental caries and obesity. ^46^ Frias-Bulhosa et al. 2015 proposed that oral hygiene habits had more statistical significance in the association between dental caries and BMI. Using multivariate analysis. the oral hygiene frequency was observed to be significantly associated with DMFT higher than zero (p = 0.041). ^47^ This is an extended link which is the link between malnutrition and oral hygiene. In more depth, Vieira, et al. 2020 found that malnutrition in children applies a negative effect on the oral cavity and a decrease in the salivary flow rate was detected with the increase in the degree of malnutrition. Diagnosing the effects of malnutrition regards the oral cavity of children is essential because it can enhance the quality of life and give them an acceptable therapy. ^48^ Besides, da Fonseca, et al. 2017 stated that malnutrition deprives the kid from the important nutrients for growth and development, including that of oral structures ***.*** Its undesirable impact is obvious in the integrity of the oral mucosa and salivary function, hence affecting the oral hygiene with great risks of dental caries due to harboring the cariogenic bacteria. ^49^

Interestingly, caries was also detected in wasted children with low BMI in our study. A Pearson correlation test was performed-in a study done by Shakya, et al. 2013 and yielded similar findings - between BMI score and dft score of the deciduous dentition. A negative association (-0.016) was observed. Likewise, the Pearson correlation test was carried out for DFT score of permanent dentition of age groups 10 and 12. A negative correlation was obtained in both age groups (−0.108 and −0.033 respectively). The finding suggested that children with less BMI score tend to have more caries affected teeth than children with normal BMI. ^50^ Yang, et al, 2015 also found an inverse relationship between body BMI and caries. ^45^ Although that study used CDC charts ^51^ to address a different parameter (BMI) to identify underweight other weight for age (the parameter used by WHO to identify underweight), yet, BMI was used to identify overweight (as WHO recommended) which had negative correlations with caries in that study. ^4^ To illustrate, Pearson's correlation between dmft/(dmft + DMFT) and BMI was significant [ *P* = 0.04 for dmft, *P* = 0.004 for (dmft + DMFT)], with R values of −0.075 and −0.104 for dmft and (dmft + DMFT), respectively. These findings revealed an inverse relationship between BMI and caries severity.

Underweight is determined by the weight for age parameter in children not by BMI except after the age of ten years at which BMI can be used as a parameter to address both underweight and wasting as WHO recommends BMI as the ideal measure after the age of 10 years due to the variable age of puberty. Tracking weight alone is not recommended. Underweight or wasting among the adolescents (10-19 y) is defined as a BMI-for age below -2 Z-scores of a reference. ^4^

Surprisingly, underweight was associated with dental caries in our findings. Goodson, et al, 2013 used weight for age WHO charts to identify underweight as our study applied. That study found reduced dental decay with an increase in the body weight. ^44^ That study enrolled children with age more than 10 years,however, it didn’t used BMI to identify the underweight in those children as WHO recommended in this age group. Still, it was found that the correlations between BMI and the percent of teeth decayed or filled were nonlinear. Therefore, more increased caries in the underweight children can be concluded.

Two explanations were suggested for this relation between low BMI or underweight and dental caries. Firstly, the uncomfortable and painful feelings resulted from the untreated dental caries prevented the kids from eating and ultimately caused wasting or underweight. Secondly, kids who are picky eaters are more probable to be served kinds of unhealthy foods to motivate their appetite, that consequently induced underweight or wasting and dental caries. ^52^

MIH is presently more often observed in the dental centers or clinics that signifies a great challenge for the dental practitioners. ^53^ The offending factors are thought to interplay during the first four years of life, and impede the maturation and/or calcification phases of amelogenesis leading to qualitative defects of enamel or hypomineralization. This can explain the association between the stunted growth and MIH in our study as causes of short stature tend to exist in the earlier years of life. ^54^ However, Owlia, et al. 2020 detected that schoolchildren with lower body height had no significant correlation or association ( *P* ˃ 0.05) with MIH. ^55^ This disagreement between our results and those findings may be due to the high prevalence of short stature in the Egyptian children. ^56, 57^

Molar Incisor Hypomineralization and Hypomineralized Second Primary Molars include qualitative structural developmental anomalies of the tooth enamel affecting the first permanent molars (and often the incisors) and the second primary molars, respectively. A putative correlation between HSPM and MIH has been stated in the literature. This may disclose the clinical significance and the importance of detecting the correlation between them in the present study with further implications of preventive interventions that could reduce the MIH complications. ^58^ In fact, HSMP could be considered a predictor of MIH. ^59^

The defective enamel seen in HSPM can also be a center of diminished caries resistance because in hypomineralized teeth, there is an increase in porosity and the rod structure is constantly disorganized. ^60^ Second primary molars erupt after the first primary ones, but a higher prevalence of dental caries has been detected in the second primary molars compared to the first primary molars, that could be clarified by the predilection of HSPM for second primary molars. Therefore, HSPM may be an important risk factor for early loss of second primary molars. ^61^ This can justify the association between HSPM and caries index in our report, the finding that is very close to Halal, et al. 2020 that observed an increased incidence of dental caries found in children with HSPM. Children with HSPM were more probable to have dental caries (OR 6.69; CI 4.5_10; p < 0.001). ^62^

The literature about MIH provides data about the relation between dental caries and MIH, and former studies showed that children with severe MIH are more likely to present further with dental caries as our study revealed. NegreBarber, et al, 2018 identified that the prevalence of caries was higher among pediatrics with severe form (60.7%) than in those with milder form (43.1%) or no molar incisor hypomineralization (45.5%). ^63^ Nevertheless, Heitmüller, et al. 2013 found no correlation between MIH and dental caries (P-values > 0.05). ^64^ Considering the malnutrition effect in our study on the oral hygiene of children, poorer oral hygiene detected in pediatrics with MIH might be an attributing risk factor for the high prevalence of caries found in children with MIH. ^65^ While a systematic review of published researches proposed an association between these two conditions, the results should, however, be interpreted with caution owing to the lack of high quality studies. ^66^ The limitations to comparing the results detected for the link between MIH and dental caries involve the different criteria used in the studies to assess the presence and severity of MIH and dental caries. What is more, no agreement was observed between our finding regarding the prevalence of MIH and Ahmad, et al. 2019 who detected low prevalence (7.57%) of MIH. ^67^ This disagreement may bring to light the comorbid effect of malnutrition in our study as it could be a considerable risk factor for MIH, hence the high prevalence of MIH in our current study- that enrolled the malnourished children- ensued.

The socioeconomic status of the study participants has been investigated. A study done by AbdElAziz and Hegazy, et al, 2012. had identified the socioeconomic risk factors for malnutrition and recommended to design the targeted preventive interventions which will be important for amelioration of the undernutrition situation. Health education towards better child nutrition attitude and practices may serve to decrease the prevalence of malnutrition. ^31^

Indeed, the socioeconomic status has many associations in our study beyond malnutrition or obesity as discussed before. To explain, it is linked to the dental abnormalities. For example, the present study showed higher percentages of caries in lower socioeconomic levels. These results agree with Wang, et al, 2017 whose study yielded lower socioeconomic levels in individuals with caries. Bivariate analysis in that study yielded a significant correlation between SES and DMFT (p˂0.05). That study suggested that high household income,high educational level, and existing in a non-agricultural community are protective factors from the dental caries. ^68^ However, the present study slightly disagrees with Oyedele, et al, 2016 whose study found that the socioeconomic level contributed no significant risk (p = 0.67) for HSPM. ^69^ This disagreement may be due that in our study, the malnutrition was an added risk factor beside the low socioeconomic level.

There are some limitations to be highlighted in this study. Firstly, not including controls of well-nourished children is considered as a limitation of the study. Not including controls of well nourished children in the study may make it difficult to detect whether the dental abnormalities are more prevalent in the malnourished children or not. However, the aim of the study was assessing the prevalence of dental abnormalities in the malnourished children, not comparing them with the well nourished ones. This may open the way for further researches aiming at addressing the difference of the prevalence of the dental abnormalities between the well nourished children and the malnourished ones. Secondly, the non-probability sampling technique (consecutive sampling method) may compromise the generalizability (the external validity of the study).

## Conclusion

To the best of our knowledge, this is the first study which can identify that children with both high and low BMI could be at risks of dental caries. Moreover, this is the first study to address the prevalence of MIH, HSPM and CI collectively in the malnourished children. In general, malnutrition could be a risk factor for dental abnormalities. Not including controls can be a limiting factor. Children with low socioeconomic levels have a greater incidence for HSPM compared to children with higher socioeconomic level. In addition, different dental abnormalities could co-exist together. Screening for HSPM, MIH and CI in malnourished children is recommended as it would be a welcome development

## Data availability

### Underlying data

Figshare. Childhood malnutrition and Hypomineralized molar defects, a cross sectional study. https://doi.org/10.6084/m9.figshare.16778557.v2. ^70^

Data are available under the terms of the Creative Commons Zero “No rights reserved” data waiver (CC0 1.0 Public domain dedication).
