# Supplementary material for: Childhood malnutrition and hypo mineralized  molar defects ;a cross sectional study, Egypt
Source: F1000Res. 2022 Feb 14;10:1307. Originally published 2021 Dec 22. [Version 2] doi: 10.12688/f1000research.74557.2 (PMC8764560; doi:10.12688/f1000research.74557.2)
Supplement: Peer review history

# Peer review: Reviewer response for version 2

**DOI:** 10.5256/f1000research.120973.r123463

**Published:** 2022-02-28

**Authors:** Huda Basaleem

**Affiliations:** 1 Community Medicine and Public Health, Faculty of Medicine and Health Sciences, University of Aden, Aden, Yemen

I read the revised manuscript and approved it.

Is the work clearly and accurately presented and does it cite the current literature?

Yes

If applicable, is the statistical analysis and its interpretation appropriate?

Yes

Are all the source data underlying the results available to ensure full reproducibility?

Yes

Is the study design appropriate and is the work technically sound?

Yes

Are the conclusions drawn adequately supported by the results?

Yes

Are sufficient details of methods and analysis provided to allow replication by others?

Yes

Reviewer Expertise:

NA

I confirm that I have read this submission and believe that I have an appropriate level of expertise to confirm that it is of an acceptable scientific standard.

# Peer review: Reviewer response for version 2

**DOI:** 10.5256/f1000research.120973.r123464

**Published:** 2022-02-14

**Authors:** Hossam Abdelatty Eid Abdelmagyd

**Affiliations:** 1 College of Dentistry, Gulf Medical University, Ajman, United Arab Emirates

The points I have raised in my review report have been addressed by authors and the edited manuscript has been approved for indexing.

Is the work clearly and accurately presented and does it cite the current literature?

Partly

If applicable, is the statistical analysis and its interpretation appropriate?

Partly

Are all the source data underlying the results available to ensure full reproducibility?

Yes

Is the study design appropriate and is the work technically sound?

Partly

Are the conclusions drawn adequately supported by the results?

Partly

Are sufficient details of methods and analysis provided to allow replication by others?

Partly

Reviewer Expertise:

NA

I confirm that I have read this submission and believe that I have an appropriate level of expertise to confirm that it is of an acceptable scientific standard.

# Peer review: Reviewer response for version 1

**DOI:** 10.5256/f1000research.78328.r117503

**Published:** 2022-01-17

**Authors:** Hossam Abdelatty Eid Abdelmagyd

**Affiliations:** 1 College of Dentistry, Gulf Medical University, Ajman, United Arab Emirates

**Abstract**

- The comment written in the results part has to be rephrased to be statically significant mentioning p-value
- Keywords: no abbreviation in keywords is recommended

**Introduction**

- Too short introduction
- Definition of malnutrition and its classifications have to be mentioned  
- Classification of age groups based on WHO has to be added

**Methods**

- Study groups did not include a control group 
- Sample size not mentioned
- Ethical approval of the institute not included
- Exclusion criteria should include children with systemic diseases related to the digestive system

**Results**

- The statistical test used for data analysis is not mentioned

**Discussion**

- The authors didn’t use numbers or percentages when comparing their study results with other studies which are very important for the quality of the discussion
- Study limitations mentioned by authors themselves need to be addressed for validation of the results and conclusion 

Is the work clearly and accurately presented and does it cite the current literature?

Partly

If applicable, is the statistical analysis and its interpretation appropriate?

Partly

Are all the source data underlying the results available to ensure full reproducibility?

Yes

Is the study design appropriate and is the work technically sound?

Partly

Are the conclusions drawn adequately supported by the results?

Partly

Are sufficient details of methods and analysis provided to allow replication by others?

Partly

Reviewer Expertise:

Oral diagnosis, oral medicine, Periodontology, Immunology, medical education

I confirm that I have read this submission and believe that I have an appropriate level of expertise to confirm that it is of an acceptable scientific standard, however I have significant reservations, as outlined above.

# Response

**Published:** 2022-02-02

**Authors:** Hoda Atef

**Affiliations:** Cairo University, Egypt

Thank you dear reviewer for your valuable comments

 Point by point response to the comments 

**1. Reviewer's comment:** In the abstract section,

- The comment written in the results part has to be rephrased to be statically significant mentioning p-value

- Keywords: no abbreviation in keywords is recommended

**Author's response: ** Done in the revised version

**2. Reviewer's comment**, In the introduction part,

- Too short introduction
- Definition of malnutrition and its classifications have to be mentioned  
- Classification of age groups based on WHO has to be added

**Author's response: ** 

- I have enriched the introduction with more information and details.
- Definitions of malnutrition and classification based on age by WHO have been addressed in the introduction with more details in the methodology as these classifications have been used in identifying malnutrition in the study group

**3. **Re** viewer's comment ** In the methods parts,

- Study groups did not include a control group 
- Sample size not mentioned
- Ethical approval of the institute not included
- Exclusion criteria should include children with systemic diseases related to the digestive system

**Author's response:**

- One of the limitations of the study is that it didn’t include control groups. More studies are needed to address the difference between the malnourished and the well- nourished children regarding the dental abnormalities.
- There is a subtitle in the method section for sample size estimation and technique.
- There is a subtitle in the method section for the ethical approval; approved by the scientific research committee and ethics of Cairo University, Faculty of Dentistry (ethical clearance number, 13321) and the study was carried out in accordance with Cairo University's laws for  human research.
- One of the exclusion criteria was children with chronic diseases which include diseases related to the digestive system  

**4. **Rev** iewer's comment, **In the result part **, **

- The statistical test used for data analysis is not mentioned

**Author's response:**

- Written in the subtitle ( data analysis ) of the method section

**5. Reviewer's comment,** in the discussion

- The authors didn’t use numbers or percentages when comparing their study results with other studies which are very important for the quality of the discussion
- Study limitations mentioned by authors themselves need to be addressed for validation of the results and conclusion 

**Author's response:**

- I have enriched the discussion by more than 30 updated references with comparing the results.
- We agree with the reviewer regards the study limitation and it was described as a limitation. Any study has its own limitations, so further studies are needed to further address the possible extended outcomes of the research.

# Peer review: Reviewer response for version 1

**DOI:** 10.5256/f1000research.78328.r117506

**Published:** 2022-01-10

**Authors:** Huda Basaleem

**Affiliations:** 1 Community Medicine and Public Health, Faculty of Medicine and Health Sciences, University of Aden, Aden, Yemen

The topic is well chosen and addressing an interesting issue.

**Title**

- Better to add Egypt (a cross-sectional study, Egypt).

**Abstract**

- Background: add “of the study” to the objective of the study.
- Methods: missing details about: sample technique (consecutive), period of the study, variables, and statistical analysis.
- Keywords: better to write the full name.

**Introduction**

 The verbs of the objectives need to be amended:

- The primary objective of the study is to estimate the prevalence……..
- The Secondary objectives involve assessing the association between HSPM, MIH, and dental caries with co- occurrence of HSBM and MIH, and determining……….

**Methods**

- This section was written in a bulleted manner. The numbers 1-6 are not needed. Re-write
- Please cite reference(s) for malnutrition definition.
- Data “were” not data “was”.

**Results**

- The 1st paragraph is not needed (it is a repetition of the methodology).
- The writing needs to be improved in many place. Avoid having small fragmented paragraphs like the description of tables in page 7.
- No need to mention the M:F ratio as the sample are equally divided by gender.
- There are 10 tables which are many for such a paper. Some tables are not needed like table 1, 3, 5 and 8. They are enough to be written as a text.
- The description of the tables is misplaced. There is a possibility to put the writing about the table immediately before the table. The description of Table 10 is currently below the table; it should be before it.
- Better to write the sample size in each title of the tables (n=54).

**Discussion**

- This section needs to be totally re-written. It is now written with similar paragraphs and lacks the necessary depth of a sound discussion.
- Instead of writing “a previous study”, it is better to indicate which study: for example by the author, country or the year, or any other identification information.

Is the work clearly and accurately presented and does it cite the current literature?

Yes

If applicable, is the statistical analysis and its interpretation appropriate?

Yes

Are all the source data underlying the results available to ensure full reproducibility?

Yes

Is the study design appropriate and is the work technically sound?

Yes

Are the conclusions drawn adequately supported by the results?

Yes

Are sufficient details of methods and analysis provided to allow replication by others?

Yes

Reviewer Expertise:

Community medicine and public health with subspecialty in nutrition

I confirm that I have read this submission and believe that I have an appropriate level of expertise to confirm that it is of an acceptable scientific standard, however I have significant reservations, as outlined above.

# Response

**Published:** 2022-02-02

**Authors:** Hoda Atef

**Affiliations:** Cairo University, Egypt

Thank you dear reviewer for your valuable comments

 Point by point response to the comments 

**1. Reviewer's comment** : In the title  section ,

- Better to add Egypt (a cross-sectional study, Egypt).

**Author's response: ** Done in the revised version

**2. Reviewer's comment:** In the abstract section,

- Background: add “of the study” to the objective of the study.

- Methods: missing details about: sample technique (consecutive), period of the study, variables, and statistical analysis.
- Keywords: better to write the full name.

**Author's response:** Done

**3.**** Reviewer's comment**, In the introduction part ,

 The verbs of the objectives need to be amended:

- The primary objective of the study is to estimate the prevalence……..
- The Secondary objectives involve assessing the association between HSPM, MIH, and dental caries with co- occurrence of HSBM and MIH, and determining……….

**Author's response:** Done

**4. **R** eviewer's comment ** In the methods parts,

- This section was written in a bulleted manner. The numbers 1-6 are not needed. Re-write
- Please cite reference(s) for malnutrition definition.
- Data “were” not data “was”.

**Author's response: **Done

**5. **Reviewe** r's comment, **In the result part **,**

- The 1st paragraph is not needed (it is a repetition of the methodology).
- The writing needs to be improved in many place. Avoid having small fragmented paragraphs like the description of tables in page 7.
- No need to mention the M:F ratio as the sample are equally divided by gender.
- There are 10 tables which are many for such a paper. Some tables are not needed like table 1, 3, 5 and 8. They are enough to be written as a text.
- The description of the tables is misplaced. There is a possibility to put the writing about the table immediately before the table. The description of Table 10 is currently below the table; it should be before it.
- Better to write the sample size in each title of the tables (n=54).

**Author's response:** Done

**6. **Revie** wer's comment ** The conclusion should have limitation and probably Recommendation.

**Author's response:** Done

**7. Reviewer's comment**, In the discussion section,

- This section needs to be totally re-written. It is now written with similar paragraphs and lacks the necessary depth of a sound discussion.
- Instead of writing “a previous study”, it is better to indicate which study: for example by the author, country or the year, or any other identification information

​​​​ **Author's response:** Done, I have rewritten the discussion section and updated it

# Peer review: Reviewer response for version 1

**DOI:** 10.5256/f1000research.78328.r117504

**Published:** 2022-01-05

**Authors:** Osama El-Asheer

**Affiliations:** 1 Pediatric Department, Faculty of Medicine, Assiut University, Assiut, Egypt

I have reviewed the document from my Egyptian colleagues with great interest. This paper is well written and addresses an important topic which is the screening of dental abnormalities in malnourished children. The article is clear and of relevance also because there is a need for clinicians to develop an awareness about the possibility of dental abnormalities in the setting of malnutrition. It is today rarely described in international medical journals and there is no sufficient researches or data describing this relationship. Furthermore, the tool respects the WHO classification of malnutrition.

 Although the nutritional assessment here in the article depended only on the anthropometric evaluation, and others tools like the biochemical or dietary assessment are not present, there are several important conclusions from this article. For example, dental caries is a morbidity that could be found in obese children, and hypo-mineralization could be a morbidity in children with short stature. However, a correlation with obesity comorbidities could have been done to disclose more significance about the relationship between malnutrition and dental abnormalities. Very important: malnutrition could even represent a risk for the co-occurrence between MIH and HSPM. Correctly the authors excluded the chronic diseases to exclude bias as the chronic course could cause organic failure to thrive.

 To improve paper readability, I suggest the following. Firstly, the discussion - although clear and focused - is better to be enriched with more related references. Secondly, the tables are better to be less, or summarized.

Is the work clearly and accurately presented and does it cite the current literature?

Yes

If applicable, is the statistical analysis and its interpretation appropriate?

I cannot comment. A qualified statistician is required.

Are all the source data underlying the results available to ensure full reproducibility?

Yes

Is the study design appropriate and is the work technically sound?

Yes

Are the conclusions drawn adequately supported by the results?

Yes

Are sufficient details of methods and analysis provided to allow replication by others?

Yes

Reviewer Expertise:

Infant and pediatric clinical nutrition

I confirm that I have read this submission and believe that I have an appropriate level of expertise to confirm that it is of an acceptable scientific standard.

# Response

**Published:** 2022-02-02

**Authors:** Hoda Atef

**Affiliations:** Cairo University, Egypt

Thank you dear reviewer for your valuable comments

 Point by point response to the comments

**1. Reviewer's comment**: Firstly, the discussion - although clear and focused - is better to be enriched with more related references

**Author's response:** Done, I have enriched the discussion section by more than 30 updated references in the revised version 

**2. Reviewer's comment:** the tables are better to be less, or summarized.

**Author's response:** Done in the result section of the revised version
